# MDM2 and P53 polymorphisms contribute together to the risk and survival of prostate cancer

**DOI:** 10.18632/oncotarget.3923

**Published:** 2015-05-18

**Authors:** Li Xue, Xiujuan Han, Rongrong Liu, Ziming Wang, Hecheng Li, Qi Chen, Peng Zhang, Zhenlong Wang, Tie Chong

**Affiliations:** ^1^ Department of Urology, The Second Affiliated Hospital, Xi'an Jiaotong University, Xian, China; ^2^ The Helmholtz Sino-German Research Laboratory for Cancer, Department of Pathology, Tangdu Hospital, The Fourth Military Medical University, Xian, China; ^3^ Department of Pathology, School of Basic Medicine, Fourth Military Medical University, Xian, Shaanxi, China

**Keywords:** MDM2 gene, p53 gene, prostate cancer, risk, survival

## Abstract

The p53 gene and MDM2 gene play critical roles in cell cycle arrest and apoptosis together. Here, we evaluated the associations of prostate cancer risk and survival with the joint effects of mdm2 and p53 polymorphisms. Totally 1,193 cases and 1,310 age frequency-matched controls were included in the study. Prostate cancer patients were followed to determine the intervals of overall survival and disease-free survival. The Pro^72^Arg Pro allele (homozygous and heterozygous) were significantly associated with prostate cancer risk with an odds ratio (OR) of 0.77 [95% confidence interval(CI), 0.64-0.93]. SNP309 T alleles were associated with a significantly decreased prostate cancer risk among Pro^72^Arg Pro alleles carriers (OR=0.79, 95% CI, 0.64-0.98). In addition, comparedwith the Pro^72^Arg Pro alleles and SNP309 G homozygous, patients carrying both SNP309 T alleles and Pro^72^Arg Arg homozygous had more favorable disease-free survival (hazard ratio [HR] = 0.59, 95% CI, 0.38-0.93). Our results indicated that SNP309 and Pro^72^Arg polymorphisms may jointly contributeto the etiology and prognosis of prostate cancer.

## INTRODUCTION

Prostate cancer is the second most frequently diagnosed cancer and the sixth leading cause of cancer-related death among males worldwide [1]. It is the most common type of cancer in men in the United States, with 186,000 new cases in 2008 and 28,600 deaths [2]. It has been recognized that prostate cancer, which is a complex and multifactorial disease, is a result of interplay between different exposures and host susceptibility. The tumor suppressor p53 pathway could prevent carcinogenesis by causing cell cycle arrest or apoptosis [3-5]. The p53 gene has a functional single nucleotide polymorphism (SNP), the G > C change at codon72 in exon 4 (Pro72Arg, rs1042522), which results in an arginine-to-proline change in the protein sequence [6]. This polymorphism is located in the proline-rich domain which is necessary for the P53 protein to fully induce apoptosis [7]. The Arg allele is significantly more efficient in inducing apoptosis, while the Pro allele appears to have a higher capacity for DNA repair and cell cycle G1 arrest [8]. It's also reported that the polymorphism of TP53 at codon 72 could influence the accumulation of mtDNA mutations [9]. Human mouse double-minute 2 protein gene (mdm2) is an important negative regulator of p53 and its over expression is associated with increased metastasis, decreased response to therapy, and poor prognosis [10-13]. A functional SNP in the mdm2 gene promoter region (SNP309, rs2279744) elevated mdm2 gene transcription under the influence of estrogens signaling and the subsequent attenuation of the p53 pathway and may represent a cancer predisposing allele [14, 15].

Given the functional relevance of p53 and mdm2 in cell-cycle control and apoptosis, the combination of these polymorphisms is expected to determine susceptibility and prognosis of the prostate cancer more accurately than alone. We hypothesized that common variants of mdm2 and p53 and their joint effects are associated with risk and survival of prostate cancer. We therefore performed genotyping analyses for SNPs of SNP309, SNP354 in mdm2 gene and Pro^72^Arg in p53 gene in a large case-control study conducted in Chinese male population.

## RESULTS

The clinical features of the 1,193 patients with prostate cancer and 1,310 control males are shown in Table 1. The mean age of the prostate cancer patients and the controls at the time that the blood was drawn was 69.5 and 70.1 years, respectively. There were no significant differences between the controls and cases with regard to age, smoking status, drinking status or BMI.

**Table 1:** Clinical characteristics of the controls and patients

Variables	Patients (n = 1,193)	Controls (n = 1,310)	*P*-value
Age at diagnosis	69.5 ±8	70.1±9	0.08
Family history			
Yes	154	30	
No	1,039	1,280	*P* < 0.001
Smoking status			
Never	915	1,041	0.091
Ever	278	269	
Drinking status			
Never	892	998	0.411
Ever	301	312	
Body mass index			
<25 kg/m2	656	721	0.654
25–29.9 kg/m2	477	537	
≥30 kg/m2	60	52	
PSA levels at diagnosis, ng/mL	20.7±6.6		
Gleason score			
2-6	620		
7	418		
8-10	155		
Clinical stage, T3%	67 (5.6%)		
Treatment			
hormonal therapy	675		
Androgen Deprivation	251		
Radiation	489		

Table 2 shows the association between SNP309 and SNP354 in mdm2 and Pro^72^Arg in p53 gene and prostate cancer risk. The distribution of genotypes for these three polymorphisms is consistent with the Hardy-Weinberg equilibrium for both cases and controls. Compared with subjects with the Pro^72^Arg/ Arg homozygous, those with the Pro^72^Arg Pro allele, including the homozygous and heterozygous categories, had showed a protective effect on prostate cancer (odds ratio [OR] = 0.77, 95% confidence interval [CI], 0.64-0.93, *P* = 5.54×10^−3^). Stratified analyses by Gleason score and clinical stage showed that no significant difference (supplementary table 1).

**Table 2:** MDM2 and p53 genotypes and prostate cancer risk

Genotype	Cases	Controls	Adjusted OR (95% CI)*
**MDM2**			
SNP309			
GG	334	356	1.00 (reference)
GT	565	602	1.00 (0.83-1.21)
TT	227	272	0.89 (0.71-1.12)
GT+TT	792	874	0.97 (0.81-1.15)
SNP354			
AA	1037	1046	1.00 (reference)
AG	40	35	1.15 (0.73-1.83)
**P53**			
P53 codon72			
Arg/Arg	339	305	1.00 (reference)
Arg/Pro or Pro/Pro	751	875	0.77 (0.64-0.93)

* Adjusting for age at diagnosis, family history, smoking status, dringk status, and BMI.

In order to evaluate the joint effect of mdm2 polymorphisms and p53Arg^72^Pro genotypes on prostate cancer risk, we performed stratification analyses by p53 Arg^72^Pro genotypes. As shown in Table 3, we found that the variant genotypes of SNP309 GT and TT were associated with a significantly decreased prostate cancer risk among carriers with p53 Pro alleles (OR = 0.79, 95% CI: 0.64-0.98, P for interaction = 0.0112). We examined the potential interactive effect between SNP354 and p53 Pro^72^Arg genotypes and no significant interaction were observed.

**Table 3:** Gene-gene interaction of MDM2 and p53 genotypes for prostate cancer risk

Genotypes							
	p53 codon72						
		Arg/Arg			CG+CC		
		Case	Control	OR(95% CI)*	Case	Control	OR(95% CI)*
MDM2 SNP 309							
GG		80	91	1.00 (reference)	244	232	1.00 (reference)
GT+TT		256	207	1.41 (0.99-1.99)	502	604	0.79 (0.64-0.98)
	***p* for interaction = 0.0112**						
MDM2 SNP 354							
AA		308	285	1.00 (reference)	687	706	1.00 (reference)
AG		13	10	1.20 (0.52-2.79)	27	24	1.16 (0.66-2.02)
	*p* for interaction = 0.9406						

* Adjusting for age at diagnosis, family history, smoking status, dringk status, and BMI.

The median follow-up time for prostate cancer patients was approximately 7 years. Table 4 presents HRs and 95% CIs of mdm2 and p53 polymorphisms after adjustment for potential confounding factors, including TNM stage, radiotherapy, and age. Overall, neither overall survival nor disease-free survival was associated with the SNP309, SNP354 or Pro^72^Arg polymorphisms (Table 4). We next addressed whether there is a joint effect of mdm2 and p53 polymorphisms on prostate cancer survival. We found a statistically significant interaction between SNP309 and Pro^72^Arg for prostate cancer disease-free survival (*P*_interaction_ = 0.0298). Compared with the Pro^72^Arg Pro alleles (homozygous and heterozygous) and SNP309 G homozygous, patients carrying both SNP309 T (homozygous and heterozygous) and Pro^72^Arg Arg homozygous had more favorable disease-free survival [hazard ratio (HR) = 0.59, 95% CI: 0.38-0.93]. These sup-group patients also had better overall survival rates, although the association was not statistically significant (HR = 0.71, 95% CI: 0.41-1.21). However, we did not find the same strong relationship between SNP354 and Pro^72^Arg polymorphisms (Table 5).

**Table 4:** Association of mdm2 and p53 genotypes and prostate cancer survival

Genotypes	Case	Overall survival		Disease-free survival	
		Events	HR (95% CI) *	Events	HR (95% CI) *
mdm2					
SNP309					
GG	334	74	1.00 (reference)	97	1.00 (reference)
GT+TT	792	169	1.03 (0.78-1.35)	210	0.93 (0.73-1.19)
GT	565	120	1.01 (0.75-1.36)	148	0.92 (0.71-1.19)
TT	227	49	1.06 (0.73-1.52)	62	0.97 (0.71-1.35)
SNP354					
AA	1037	220	1.00 (reference)	281	1.00 (reference)
AG	40	11	1.20 (0.65-2.22)	11	1.07 (0.58-1.95)
P53					
SNP codon72					
Arg/Arg	339	72	1.00 (reference)	97	1.00 (reference)
Arg/Pro or Pro/Pro	522	107	1.06 (0.78-1.43)	132	0.96 (0.74-1.26)

* Adjusting for age at diagnosis, family history, PSA levels at diagnosis, PSA recurrence, Gleason score, clinical stage, and treatment.

**Table 5:** Gene-gene interaction of mdm2 and p53 genotypes in relation to the prostate cancer survival

Genotypes	Overall survival						Disease-free survival					
	P53 codon72						P53 codon72					
	Arg/Arg			Arg/Pro or Pro/Pro			Arg/Arg			Arg/Pro or Pro/Pro		
	Case	Events	HR (95% CI)*	Case	Events	HR (95% CI)*	Case	Events	HR (95% CI)*	Case	Events	HR (95% CI)*
*mdm2 SNP 309*												
GG	80	21	1.00 (reference)	244	51	1.00 (reference)	80	30	1.00 (reference)	244	66	1.00 (reference)
GT+TT	256	51	0.71 (0.41-1.21)	502	109	1.18 (0.84-1.66)	256	66	0.59 (0.38-0.93)	502	134	1.08 (0.80-1.46)
	*p* for interaction=0.1353,						***p* for interaction=0.0298**					
*mdm2 SNP 354*												
AA	308	64	1.00 (reference)	687	145	1.00 (reference)	308	89	1.00 (reference)	687	181	1.00 (reference)
AG	13	4	1.37(0.48-3.90)	27	7	1.20 (0.56-2.58)	13	4	1.38(0.50-3.85)	27	7	0.97 (0.45-2.07)
	*p* for interaction=0.8222						*p* for interaction=0.5864					

* Adjusting for age at diagnosis, family history, PSA levels at diagnosis, PSA recurrence, Gleason score, clinical stage, and treatment.

## DISCUSSION

In the present study we examined whether genetic polymorphisms in p53 and mdm2, alone or in combination, are associated with the risk and survival of prostate cancer in a Chinese population. Our results demonstrate that Pro^72^Arg Pro alleles were significantly associated with decreasing prostate cancer risk. A joint protective effect of Pro^72^Arg Arg alleles and SNP309 T alleles were detected. Furthermore, we found a significant gene-gene interaction between SNP309 and Arg^72^Pro variants in relation to survival of prostate cancer.

The Arg^72^Pro polymorphism in p53 gene was well characterized in both functional analyses and association studies [16-21]. Our data suggested that the Pro alleles were potent genetic protective factor for prostate cancer. The findings are supported by the earlier described functional significance of the Pro^72^Arg polymorphism and studies for association of Pro^72^Arg Pro with prostate cancer risk [22-25]. We did not find SNP309 or SNP354 polymorphism alone to be associated with prostate cancer risk. In consistent, null association between SNP309 and prostate cancer were also observed in other population [26]. No study has examined the joint effect of polymorphisms in mdm2 and p53 genes in prostate cancer risk. Interestingly, we found a significant joint protective effect of Pro^72^Arg/Pro alleles and SNP309 T alleles in Chinese population. The joint effect between these two genotypes is biologically plausible. MDM2 and P53 act in the same causal pathway for carcinogenesis [27, 28]. MDM2 down regulates P53 activity by binding it directly and forming the MDM2-P53 complex, which results in ubiquitination and proteasome degradation of P53 through the E3 ubiquitin ligase activity of MDM2 [29]. If a cell carries functional polymorphisms in both genes that diminish the expression of MDM2 and heighten the function of P53, a gene-gene joint protective effect would be expected [29]. It has been shown that the Pro^72^Arg Pro allele (homozygous and heterozygous) was positively associated with the transcriptional activity of p53 gene *in vitro* [8]. The SNP309 G homozygous result in overexpression of MDM2 protein and thus inhibits chromatin-bound P53 from activating the transcription of its target genes [14, 30]. In this regard, one may expect that individuals with the Pro^72^Arg Pro alleles (homozygous and heterozygous) and SNP309 T alleles (homozygous and heterozygous) are less susceptible to cancer.

Another intriguing observation evident from this study is that patients carrying both SNP309 T alleles (homozygous and heterozygous) and Pro^72^Arg Arg homozygous had more favorable disease-free survival. This result is supported by an *in vitro* study, which showed that after treatment with etoposide to induce DNA damage, which activates the p53 pathway, significant death was observed in cells with the SNP309 T homozygous but not in cells with the SNP309 G homozygous [14]. Moreover, Pro^72^Arg Arg allele have been shown to induce apoptosis more efficiently than Pro allele, which may also accelerate the apoptosis of tumor cell [4, 31-33]. Therefore, the coexistence of SNP309 T alleles (homozygous and heterozygous) and Pro^72^Arg Arg homozygous is expected to be associated with a favor prognosis. In addition to altering tumor development, the Pro^72^Arg polymorphism may alter the sensitivity of tumors to chemotherapeutic agents, Pro^72^Arg Arg homozygous might be predicted to respond more favorably to radiation or chemotherapy.

Strengths of this study include the population-based study design and a high response rate, which minimized potential selection bias. The detailed exposure information collected in the study enabled an evaluation of gene-gene interactions. Information on cancer characteristics and treatment was obtained from the vast majority of patients, allowing an evaluation of possible effect modifications. There are also a few limitations that must be considered in evaluating these results. As mentioned above, the small sample size used for some of the stratified analyses is a limitation, resulting in unstable risk estimates and insufficient statistical power for interaction tests.

In summary, our results provide evidence that the p53 Pro^72^Arg Pro allele was a protective factor for prostate cancer. Pro^72^Arg Pro allele plus SNP309 T allele were associated with a decreased prostate cancer risk. In addition, SNP309 T allele and Pro^72^Arg Arg allele had a joint effect of favor disease-free survival in prostate cancer patients, and the association with survival seemed to be independent from other clinical prognostic factors such as cancer stage.

## MATERIALS AND METHODS

### Study population

The study protocol was approved by committees of relevant institutes for the use of human subjects in research. All participants gave written informed consent. All the data of our study were stored in publicly available resources of The Second Affiliated Hospital of Xi'an Jiaotong University and available for related researchers by request. Totally this study included 1,459 men (age ranged from 39 to 87) and diagnosed with prostate cancer through a rapid case-ascertainment system using specimens from prostatic needle biopsies from Tangdu hospital, Xijing hospital and the Second Affiliated Hospital of Xi'an Jiaotong University,. A histopathological diagnosis was made by an experienced pathologist. The histological grading of the biopsy specimens was performed using Gleason's system by the same pathologist.

Meanwhile, 1,556 controls were identified and frequency matched to the expected age distribution of cases by 5-year age groups. A structured questionnaire was used to elicit detailed information on demographic factors. Blood samples were collected from 1,193 (82%) cases and 1,310 (84%) controls and used in this study for genotyping assays. Prostate cancer patients were followed for cancer recurrence and mortality by using a combination of two active follow-up surveys and record linkage to the registry of death certificates.

### Genotyping and quality control

Genotyping for SNP309 (rs2279744), SNP354 (rs769412) and Pro^72^Arg (rs1042522) was performed using the Affymetrix MegAllele Targeted Genotyping System (Affymetrix, Santa Clara, CA) according to the Affymetrix's protocol. Blinded (*n* = 39) and HapMap samples (*n* = 12) were also included with the genotyping, consistency rates averaged 99.6%. The consistence rate for the quality control samples was 99.88%.

### Statistical analyses

All statistical analyses were conducted with SAS version 9.2 (SAS Institute Inc.). All statistical tests were 2-tailed, and *P* < 0.05 was interpreted as statistically significant unless otherwise indicated. Multivariable logistic regression was used to estimate odds ratios (OR) and 95% confidence intervals (95% CI) for risk of prostate cancer, while adjusting for the confounders including age at diagnosis, family history, smoking status, drink status, and BMI. The Cox proportional hazard models were applied to evaluate hazard ratios (HRs) for the association of mdm2 and p53** polymorphisms with the overall survival (OS) and disease-free survival (DFS), adjusting for age at diagnosis, family history, PSA levels at diagnosis, PSA recurrence, Gleason score, clinical stage, and treatment.

## SUPPLEMENTARY MATERIAL TABLE



## Abbreviations

CI: confidence interval
DFS: disease-free survival
HR: hazard ratio
OR: odds ratio
OS: overall survival
SNP: single nucleotide polymorphism
