# Supplementary material for: MDM2 and P53 polymorphisms contribute together to the risk and survival of prostate cancer
Source: Oncotarget. 2015 May 18;7(22):31825–31. doi: 10.18632/oncotarget.3923 (PMC5077979; doi:10.18632/oncotarget.3923)
Supplement: Supplementary file 1 [file oncotarget-07-31825-s001.pdf]

# MDM2 and P53 polymorphisms contribute together to the risk and survival of prostate cancer

**Supplementary Material**

**Supplementary table 1 stratified analyses for associations between MDM2 and p53 genotypes and prostate cancer risk by Gleason score and clinical stage**

| | Adjusted OR (95% CI)* | | | Adjusted OR (95% CI)* | |
|---|---|---|---|---|---|
| Genotype | Gleason score (2-6) | Gleason score (7) | Gleason score (8-10) | Stage T1 | Stage T2 |
| **MDM2** | | | | | |
| SNP309 | | | | | |
| GG | 1.00 (reference) | 1.00 (reference) | 1.00 (reference) | 1.00 (reference) | 1.00 (reference) |
| GT+TT | 0.97 (0.78-1.19) | 0.95 (0.75-1.22) | 0.97 (0.67-1.39) | 0.96 (0.77-1.23) | 0.97 (0.79-1.18) |
| SNP354 | | | | | |
| AA | 1.00 (reference) | 1.00 (reference) | 1.00 (reference) | 1.00 (reference) | 1.00 (reference) |
| AG | 1.15 (0.70-1.89) | 1.13 (0.68-1.90) | 1.15 (0.60-2.21) | 1.13 (0.69-1.89) | 1.15 (0.71-1.87) |
| **P53** | | | | | |
| P53 codon72 | | | | | |
| Arg/Arg | 1.00 (reference) | 1.00 (reference) | 1.00 (reference) | 1.00 (reference) | 1.00 (reference) |
| Arg/Pro or Pro/Pro | **0.73 (0.59-0.91)** | **0.77 (0.60-0.99)** | 0.78 (0.54-1.11) | **0.77 (0.60-0.97)** | **0.76 (0.61-0.92)** |

* Asjusting for age at diagnosis, family history, smoking status, dringk status, and BMI
